# The use of biologic medications for the treatment of cutaneous immune-related adverse events secondary to immune checkpoint inhibitors: A single-institution real-life study

**DOI:** 10.1016/j.jdcr.2023.09.041

**Published:** 2023-11-02

**Authors:** Jade N. Young, Hannah Verma, Nour El Kashlan, Dina Poplausky, Angela J. Lamb, Emma Guttman-Yassky, Nicholas Gulati

**Affiliations:** Department of Dermatology, Icahn School of Medicine at Mount Sinai, New York, New York

**Keywords:** biologics, cirAE, cutaneous immune-related adverse event, immune checkpoint inhibitor, immunotherapy, oncodermatology

Immune checkpoint inhibitors (ICIs) induce antitumor effects by enhancing the host immune system to eliminate cancer cells.^1^ Because of nonspecific immune activation, ICIs cause immune-related adverse events (irAEs), including cutaneous irAEs (cirAEs), which affect approximately 40% of patients.^2^ CirAEs are associated with increased progression-free survival, but can negatively impact patient quality of life or necessitate ICI discontinuation.^3^ CirAEs typically occur within 4 to 8 weeks of ICI initiation and can occur at any point during or after treatment.^2^^,^^4^ Despite efforts to determine guidelines, the management of cirAEs is currently not standardized.^5^ Given the safety and efficacy of many biologics, their potential for managing cirAEs merits investigation. Recent reports have highlighted successful treatment of various cirAE morphologies using biologics with no worsening of tumor outcomes.^5^ However, to our knowledge, no studies to date have systematically evaluated prescribing practices of biologics for cirAEs.

We performed a retrospective chart review via prescription drug codes of patients at our institution (6 sites and 44 board-certified dermatologists) treated with both a biologic and an ICI between March 25, 2011 (date of first ICI US Food and Drug Administration approval) and January 12, 2023. Patients were identified using prescription drug codes. Biologics included dupilumab, tralokinumab, risankizumab, guselkumab, tildrakizumab, brodalumab, secukinumab, ixekizumab, bimekizumab, ustekinumab, spesolimab, adalimumab, etanercept, infliximab, golimumab, certolizumab, canakinumab, rilonacept, intravenous immunoglobulin, rituximab, obinutuzumab, omalizumab, and belimumab. ICIs included nivolumab, pembrolizumab, cemiplimab, atezolimumab, durvalumab, avelumab, ipilimumab, and relatlimab.

A total of 111 patients were identified. Of these, 7 patients received biologics for cirAE treatment, including risankizumab, dupilumab, and ustekinumab, either concurrently or after discontinuation of ICI (Table I). One patient received dupilumab for an eczematous reaction and later received risankizumab for psoriasis. No patients received dual biologic therapy. ICIs included pembrolizumab (*n* = 5), atezolizumab (*n* = 1), and nivolumab (*n* = 1). CirAEs were documented as psoriasiform (*n* = 3), eczematous transitioning to psoriasiform (*n* = 1), lichenoid (*n* = 1), eczematous (*n* = 1), and bullous pemphigoid (*n* = 1) (Table I). The only 2 adverse events, conjunctivitis and dry eyes, occurred because of dupilumab. Dermatologic evaluation occurred a median of 24 (range 0-124) days after cirAE development. Two patients were evaluated initially by dermatologists outside of the system for exacerbation of their preexisting psoriasis. Biologic initiation occurred a median of 75 (range 48-798) days from cirAE development, with a median treatment duration of 127 (range 92-2311) days. The median follow-up period postbiologic initiation was 104 (range 78-2302) days. Notably, 6 of 7 patients are alive and without cancer progression as evidenced by imaging or in remission at last follow-up. All patients significantly improved on biologic therapy.

**Table I:** Cutaneous immune-related adverse events from various immune checkpoint inhibitors treated with biologics

Immune checkpoint inhibitor	cirAE (common terminology criteria for adverse events grade)∗	Time to dermatologic evaluation (d)	Time to diagnosing cirAE (d)†	ICI stopped	Biologic treatment	Coadministered with ICI? (d of overlap)	Time to biologic initiation from cirAE (d)	Biologic treatment duration (d)	Follow-up time since biologic initiation (d)‡	Failed systemic medications	Concomitant cirAE medications	Cancer type	Cancer status while on biologic
Pembrolizumab	Psoriasis (2)	41	105	No	Risankizumab	No	757	127	98	Acitretin	None	Recurrent ovarian	Stable
Pembrolizumab	Psoriasis (2)	0§	149	Yes	Ustekinumab	No	47	2311	2302	Doxycycline	Topical steroids	Melanoma	Remission
Nivolumab	Psoriasis(2)‖	0§	28	Yes	Ustekinumab	No	98	601	601	Prednisone	None	Hepatocellular carcinoma	Progression, patient passed away after 601 d on biologic
Pembrolizumab	Eczema that converted to psoriasis(2)‖	124	609	No	Dupilumab, then risankizumab for psoriasis	Yes (688)	21	724	709	Prednisone	Topical steroids	Hepatocellular carcinoma	Stable
Pembrolizumab	Lichen planus (2)	24	136	Yes¶	Dupilumab	No	73	114	104	None	Topical steroids	Breast cancer	Stable
Atezolizumab	Eczema (2)‖	35	397	Yes	Dupilumab	Yes (11)	80	118	102	Prednisone	Prednisone (for myositis), ruxolitinib 1.5% cream	Small cell lung cancer	Stable
Pembrolizumab	Bullous pemphigoid (3)	5	1097	Yes¶	Dupilumab	No	48	92	78	Doxycycline	Ruxolitinib 1.5% cream	Melanoma/urothelial carcinoma	Remission/stable

*cirAE*, Cutaneous immune-related adverse event; *ICI*, immune checkpoint inhibitor.

∗ Psoriasiform and lichenoid cirAEs were graded using the common terminology criteria for adverse events (CTCAE) category “other skin and subcutaneous disorder” due to lack of specific categories, eczematous cirAEs were graded using the “eczema” category, and the bullous pemphigoid cirAE was graded using the “bullous dermatitis” category.

† Calculated as days from ICI initiation to cirAE presentation.

‡ Follow-up time in days calculated from biologic initiation date to date of most recent follow-up with oncology or dermatology.

§ Exacerbation was initially noted following evaluation by outside dermatologists whom the patients followed with for preexisting psoriasis.

‖ Biopsy-confirmed.

¶ Discontinued for reasons not related to cirAE.

Considering the prevalence of ICI and biologic use (4432 and 38,385 patients, respectively, within the study period at our institution), our cohort reveals that utilization of biologics for cirAE treatment is rare. We expect similar prescribing practices at other institutions given that literature on biologics use for cirAEs is limited to case reports, but this warrants further study.

The exact pathogenesis of cirAEs remains under investigation. However, one study has demonstrated that ICIs generate immune disruptions similar to the spontaneous versions of these diseases. For example, bullous pemphigoid can be induced by disrupting the skin immune barrier through trauma, allowing for exposure of self-antigens to self-reactive lymphocytes. ICIs similarly modulate the immune response through the dampening of negative checkpoints and the promotion of self-reactive lymphocytes.^6^ Such as spontaneous, non–ICI-related psoriasis, the T helper-17 pathway has been implicated in ICI-related psoriasis.^7^ This suggests that biologics acting on this pathway, including ustekinumab and risankizumab, are potential therapeutic options. Although the mechanism by which each biologic treats a given cirAE remains unclear, it can be hypothesized that it is similar to the corresponding spontaneous skin disease. As translational studies enhance our understanding of cirAE pathogenesis, implementation of targeted immunomodulation using biologics will likely increase.

There was a lengthy latency period from cirAE diagnosis to biologic initiation, and minimal overlap time between ICI and biologic. This may be because of step therapy with topical treatments and oral corticosteroids, delays in dermatologic evaluation, or safety concerns regarding concurrent use of biologics and ICIs. Further investigation of biologic use in this population could reduce this latency period and potentially reduce the rate of ICI discontinuation. However, a larger cohort is required to determine if biologic initiation in this population can reduce the rate of ICI discontinuation. Notably, 6 of 7 patients in this cohort remained stable or in remission from a cancer perspective. Further studies are needed to confirm the presented findings and optimize biologic use in this population. In addition, this investigation reveals that there are current gaps in the literature regarding long-term safety and efficacy data for biologics and cirAEs. Our findings suggest that the biologic agents discussed in this manuscript have been slowly incorporated into clinical practice for cirAEs, and thus available follow-up time is currently minimal. Also, further mechanistic research is required to determine the immunopathogenesis of and thus appropriate biologic treatment for cirAEs. Although limited to a single institution, we provide promising preliminary data on the efficacy and oncologic safety of biologics to treat cirAEs. Dermatologists can play an integral role in cirAE management by leveraging the biologics they routinely use in clinical practice.

## Conflicts of interest

Dr Guttman-Yassky is an employee of Mount Sinai and has received research grants (paid to the institution) from Boehringer Ingelheim, Leo Pharma, Pfizer, Cara Therapeutics, UCB, Kyowa Kirin, RAPT, Amgen, GSK, Incyte, Sanofi, Bristol Meyers Squibb, Aslan, Regeneron, Anaptysbio, Concert, and Janssen and/or is a consultant to AbbVie, Almirall, Amgen, Aslan Pharmaceuticals, AstraZeneca, Biolojic Design, Boehringer Ingelheim, Bristol Meyers Squibb, Cara Therapeutics, Connect Pharma, DBV Technologies, Eli Lilly, EMD Serono, Evidera, Galderma, Gate Bio, Genentech, Incyte, Inmagene, Janssen Biotech, Kyowa Kirin, Leo Pharma, Merck, Pfizer, Q32 Bio, RAPT, Regeneron, Sanofi, SATO, Siolta, Target, UCB, Ventyx. All other authors have no conflicts of interest to declare.
